# Creolin (Pearson) in Arthritic Synovitis

**Published:** 1902-02

**Authors:** George Masgana

**Affiliations:** Owensboro, Ky.


### CREOLIN (PEARSON) IN ARTHRITIC SYNOVITIS.

By George Masgana, V.S.,
OWENSBORO, KY.

Case No. 1. Black mare, seven years old, seen first on August 29, 1901. Synovitis of tarsus following a kick from a mule two weeks before seen. Hock swollen; great tumefaction of surrounding parts; open wound, discharging an ill-smelling synovia; unable to bear any weight on the injured limb; temperature, 103° F.

*Treatment.* Opened the wound more freely, injected a 20 per cent. solution of creolin, and applied a hot bath of the same strength solution for fifteen minutes three times daily. Dressed the wound with a powder, composed of iodoform, boric acid, and starch, with an oakum-pad and bandage. August 30th, no fever, no smell to pus. On the 31st the mare began to stand on the leg; purulent synovial discharge almost ceased. On the eighth day all discharge ceased, and on September 9th the mare was driven to a buggy, showing no perceptible lameness.

Case No. 2. First seen September 2, 1901; six-months-old colt; seat of synovitis, the hock-joint; time occurring, not known; the colt found by owner walking on three legs in the field. The joint was open, running freely, and the discharge very fetid in odor; articulation much swollen. The same treatment applied, save the bandaging, as the colt was too wild and restless. At the end of three days the colt was using the limb, and on the seventh day the synovial discharge had ceased, and when on the pasture field with the dam showed very little lameness.

Case No. 3. September 2d; bay gelding used in livery work; synovitis arthritis of the carpal joint, the result of a kick received three months before, but was kept at work more or less since the injury. Very lame; extensive swelling; wound discharging freely a foul-smelling, purulent synovia; temperature, 102° F. The same treatment was applied, and one week later the discharge had entirely ceased, lameness gone, the opening closed, swelling reducing. No slings were used in any of these cases.

